# Supplementary material for: Addressing benchmarking gaps in large language models for health and medicine with dynamic red-teaming
Source: Nat Health. 2026 Jul 15;1(11):1254–70. doi: 10.1038/s44360-026-00152-8 (PMC13727685; doi:10.1038/s44360-026-00152-8)
Supplement: Supplementary file 2 [file 44360_2026_152_MOESM2_ESM.pdf]

nature portfolio

Corresponding author(s): Jiazhen Pan

Last updated by author(s): May 8, 2026

Reporting Summary

Nature Portfolio wishes to improve the reproducibility of the work that we publish. This form provides structure for consistency and transparency in reporting. For further information on Nature Portfolio policies, see our Editorial Policies and the Editorial Policy Checklist.

Statistics

For all statistical analyses, confirm that the following items are present in the figure legend, table legend, main text, or Methods section.

n/a	Confirmed	
☐	☒	The exact sample size (*n*) for each experimental group/condition, given as a discrete number and unit of measurement
☐	☒	A statement on whether measurements were taken from distinct samples or whether the same sample was measured repeatedly
☐	☒	The statistical test(s) used AND whether they are one- or two-sided<br>*Only common tests should be described solely by name; describe more complex techniques in the Methods section.*
☐	☒	A description of all covariates tested
☐	☒	A description of any assumptions or corrections, such as tests of normality and adjustment for multiple comparisons
☐	☒	A full description of the statistical parameters including central tendency (e.g. means) or other basic estimates (e.g. regression coefficient) AND variation (e.g. standard deviation) or associated estimates of uncertainty (e.g. confidence intervals)
☐	☒	For null hypothesis testing, the test statistic (e.g. *F*, *t*, *r*) with confidence intervals, effect sizes, degrees of freedom and *P* value noted<br>*Give P values as exact values whenever suitable.*
☒	☐	For Bayesian analysis, information on the choice of priors and Markov chain Monte Carlo settings
☐	☒	For hierarchical and complex designs, identification of the appropriate level for tests and full reporting of outcomes
☐	☒	Estimates of effect sizes (e.g. Cohen's *d*, Pearson's *r*), indicating how they were calculated

Our web collection on statistics for biologists contains articles on many of the points above.

Software and code

Policy information about availability of computer code

Data collection	The data used in this study is either open sourced or curated from our side. We downloaded the open-sourced data directly and did not use any software.
Data analysis	We use Python 3.11 to curate the data.

For manuscripts utilizing custom algorithms or software that are central to the research but not yet described in published literature, software must be made available to editors and reviewers. We strongly encourage code deposition in a community repository (e.g. GitHub). See the Nature Portfolio guidelines for submitting code & software for further information.

Data

Policy information about availability of data

All manuscripts must include a data availability statement. This statement should provide the following information, where applicable:

- Accession codes, unique identifiers, or web links for publicly available datasets
- A description of any restrictions on data availability
- For clinical datasets or third party data, please ensure that the statement adheres to our policy

All the data used in this work are open-sourced:

- MedQA: https://huggingface.co/datasets/bigbio/med_qa

- Healthbench: https://huggingface.co/datasets/openai/healthbench
- Privacy and bias dataset: https://huggingface.co/datasets/JZPeterPan/DAS-Mediacal-Red-Teaming-Data
- Hallucination dataset: https://daneshjoulab.github.io/Red-Teaming-Dataset/

Research involving human participants, their data, or biological material

Policy information about studies with human participants or human data. See also policy information about sex, gender (identity/presentation), and sexual orientation and race, ethnicity and racism.

Reporting on sex and gender	We did not carry out human study experiments in this work.
Reporting on race, ethnicity, or other socially relevant groupings	N.A.
Population characteristics	N.A.
Recruitment	N.A.
Ethics oversight	N.A.

Note that full information on the approval of the study protocol must also be provided in the manuscript.

Field-specific reporting

Please select the one below that is the best fit for your research. If you are not sure, read the appropriate sections before making your selection.

☐ Life sciences ☒ Behavioural & social sciences ☐ Ecological, evolutionary & environmental sciences

For a reference copy of the document with all sections, see nature.com/documents/nr-reporting-summary-flat.pdf

Behavioural & social sciences study design

All studies must disclose on these points even when the disclosure is negative.

Study description	This is a quantitative computational audit/red-teaming study of medical large language models (LLMs), with qualitative examples used only to illustrate representative failure modes. We introduced Dynamic, Automatic and Systematic (DAS) red-teaming, an automated framework for stress-testing medical LLMs across four safety-relevant axes: robustness, privacy, bias/fairness, and hallucination/factual inaccuracies. The study evaluated proprietary, open-source, and medical-domain LLMs using curated clinical benchmark prompts, dynamically generated adversarial prompt mutations or disguises, and automated or expert-validated adjudication of model outputs. No patients were recruited and no clinical intervention was performed; human experts were involved only in prompt/rubric validation and adjudication of model outputs.
Research sample	The research sample consisted of LLMs and their generated responses, not human participants. We evaluated a panel of contemporary proprietary, open-source, and medical-domain LLMs; o3-mini was included only in the robustness audit. The prompt-level samples included MedQA items for structured medical question answering, 192 curated HealthBench Consensus cases for open-ended clinical reasoning, 81 privacy scenarios spanning common HIPAA/GDPR-relevant disclosure risks, 415 bias/fairness multiple-choice clinical scenarios, and 260 hallucination-detection cases consisting of 131 hallucination-positive and 129 negative/near-ideal examples. Protected demographic, linguistic, emotional, and socioeconomic attributes were used as controlled prompt perturbations in the bias/fairness audit, not as characteristics of recruited participants.
Sampling strategy	LLMs were selected purposively to cover widely used proprietary models, open-source models, and medical-specialized models available at the time of evaluation, rather than by random sampling. Prompt samples were drawn from established public or expert-vetted medical datasets and newly curated clinician-authored scenarios. For HealthBench, 200 Consensus cases were randomly selected and then filtered by two physicians for clear, medically relevant binary rubrics, yielding 192 cases. The bias/fairness set comprised 415 questions, including 304 adapted from a public medical-bias dataset and 111 authored or refined by licensed clinicians. The privacy set comprised 81 scenarios constructed to cover eight common privacy-violation modes. The hallucination set was balanced at 260 cases, with 131 high-confidence positive hallucination examples and 129 negative near-ideal examples. No formal statistical power calculation was performed; sample sizes were chosen to provide broad coverage of the intended safety axes while keeping dynamic multi-model API evaluation feasible.
Data collection	Model responses were collected by submitting original and adversarially modified clinical prompts to the target LLM APIs or open-source checkpoints. For robustness, MedQA questions first established baseline correct answers and were then subjected to dynamic mutations using a rule-constrained orchestrator and predefined mutation tools. HealthBench prompts were evaluated using physician-reviewed rubrics and adversarial perturbations adapted to open-ended clinical reasoning. Privacy prompts were tested with and without explicit privacy-warning conditions and with privacy-disguise agents. Bias/fairness prompts were tested before and after controlled identity, linguistic, emotional, and cognitive-bias perturbations. Hallucination responses were evaluated using a

	multi-agent hallucination detector organized around clinically meaningful error categories. Board-certified physicians performed blinded adjudication for selected mutation-validity checks and for validation of automated judges.
Timing	Model evaluations were conducted during the study period in which the tested model versions/checkpoints were available. Because LLM APIs and checkpoints can change over time, the specific model versions/checkpoints were recorded, and additional stability-analysis runs were timestamped. The stability-analysis logs span June 2025 to January 2026. The study did not involve participant recruitment, longitudinal follow-up, or cohort-based data collection periods.
Data exclusions	Data exclusions were based on predefined methodological criteria rather than on whether results supported the study conclusions. For robustness-HealthBench, 8 of 200 randomly selected Consensus cases were excluded because no clear medically relevant binary rubric remained after physician review, leaving 192 cases. For hallucination detection, 19 ambiguous or weakly labelled positive examples were removed after additional physician review, leaving 131 high-confidence positive samples. For robustness-MedQA, only questions answered correctly in the baseline round were used for subsequent adversarial stress testing, by design, to isolate failures of robustness from failures of baseline medical knowledge. For bias/fairness, emotional manipulation was not applied to questions originally involving psychiatric conditions to avoid confounding. Mutation tools were skipped when inapplicable to a given prompt, such as physiological-impossibility mutations for prompts without numeric clinical values.
Non-participation	Not applicable. The study did not recruit human participants, patients, or survey respondents.
Randomization	Randomization of human participants was not applicable because the study did not involve recruited participants or intervention groups.

Reporting for specific materials, systems and methods

We require information from authors about some types of materials, experimental systems and methods used in many studies. Here, indicate whether each material, system or method listed is relevant to your study. If you are not sure if a list item applies to your research, read the appropriate section before selecting a response.

Materials & experimental systems

n/a	Involved in the study
☒	☐ Antibodies
☒	☐ Eukaryotic cell lines
☒	☐ Palaeontology and archaeology
☒	☐ Animals and other organisms
☒	☐ Clinical data
☒	☐ Dual use research of concern
☒	☐ Plants

Methods

n/a	Involved in the study
☒	☐ ChIP-seq
☒	☐ Flow cytometry
☒	☐ MRI-based neuroimaging
