# Supplementary material for: Addressing benchmarking gaps in large language models for health and medicine with dynamic red-teaming
Source: Nat Health. 2026 Jul 15;1(11):1254–70. doi: 10.1038/s44360-026-00152-8 (PMC13727685; doi:10.1038/s44360-026-00152-8)
Supplement: Supplementary file 3 [file 44360_2026_152_MOESM3_ESM.pdf]

nature portfolio

Peer Review File

Dynamic Red-teaming Reveals a Benchmarking Gap in Large Language Models for Health and Medicine

Corresponding Author: Dr Jiazhen Pan

This manuscript has been previously reviewed at another journal. This document only contains information relating to versions considered at Nature Health.

This file contains all reviewer reports in order by version, followed by all author rebuttals in order by version.

Version 0:

Reviewer comments:

Reviewer #1

(Remarks to the Author)
This revised manuscript presents a substantially improved version. The central finding—a profound "Benchmarking Gap" between static performance and dynamic reliability—is well supported and clinically relevant. It might be worth directly commenting on the recently released paper in Science on medical reasoning (https://www.science.org/doi/10.1126/science.adz4433) since this work seems to indicate that, in fact, this does not seem to be the case for medical models. The revision has effectively addressed the major concerns raised by all three prior reviewers: the HealthBench extension addresses the MedQA format limitation and provides evidence of generalizability to open-ended clinical tasks; the expanded physician adjudication and cross-vendor judge analysis substantially strengthen the validation of the LLM-as-a-Judge methodology. Consider including code/data availability statements (maybe I missed?). Also, the claim that this approach is "Goodhart" resistant is quite strong and not adequately supported, please considering tempering this.

(Remarks on code availability)
Solid work and functional. Paper needs availability statement though.

Open Access This Peer Review File is licensed under a Creative Commons Attribution 4.0 International License, which permits use, sharing, adaptation, distribution and reproduction in any medium or format, as long as you give appropriate credit to the original author(s) and the source, provide a link to the Creative Commons license, and indicate if changes were made.
In cases where reviewers are anonymous, credit should be given to 'Anonymous Referee' and the source.
The images or other third party material in this Peer Review File are included in the article's Creative Commons license, unless indicated otherwise in a credit line to the material. If material is not included in the article's Creative Commons license and your intended use is not permitted by statutory regulation or exceeds the permitted use, you will need to obtain permission directly from the copyright holder.
To view a copy of this license, visit https://creativecommons.org/licenses/by/4.0/

Rebuttal Letter

DAS Red Teaming Authors

1 Reviewer #1 (Remarks to the Author)

R1.1 *This revised manuscript presents a substantially improved version. The central finding—a profound "Benchmarking Gap" between static performance and dynamic reliability—is well supported and clinically relevant. It might be worth directly commenting on the recently released paper in Science on medical reasoning (https://www.science.org/doi/10.1126/science.adz4433) since this work seems to indicate that, in fact, this does not seem to be the case for medical models. The revision has effectively addressed the major concerns raised by all three prior reviewers: the HealthBench extension addresses the MedQA format limitation and provides evidence of generalizability to open-ended clinical tasks; the expanded physician adjudication and cross-vendor judge analysis substantially strengthen the validation of the LLM-as-a-Judge methodology. Consider including code/data availability statements (maybe I missed?). Also, the claim that this approach is "Goodhart" resistant is quite strong and not adequately supported, please considering tempering this.*

A1.1 We thank Reviewer 1 for the positive feedback. We now explicitly mention the suggested Science paper Brodeur et al. (2026) in the Introduction Section by "*Most recently, Brodeur et al. reported that an LLM outperformed physician baselines across a suite of static physician reasoning tasks Brodeur et al. (2026). ...... Yet such results, however impressive, remain rooted in controlled, text-based, and largely static evaluations. We argue that static benchmarks are an insufficient and often misleading proxy for trustworthiness in health and medical applications for three reasons.*" Further, we discard the claim of "*Goodhart-resistant*" in the main text.

R1.2 *Remarks on code availability. Solid work and functional. Paper needs availability statement though.*

A1.2 We now added the Coda and Data Availability section. We also added the public GitHub code link and public dataset links in the main text.

References

Peter G Brodeur, Thomas A Buckley, Zahir Kanjee, Ethan Goh, Evelyn Bin Ling, Priyank Jain, Stephanie Cabral, Raja-Elie Abdulnour, Adrian D Haimovich, Jason A Freed, et al. 2026. Performance of a large language model on the reasoning tasks of a physician. *Science* 392, 6797 (2026), 524–527.
